# Deciphering the impact of circRNA-mediated autophagy on tumor therapeutic resistance: a novel perspective

**DOI:** 10.1186/s11658-024-00571-z

**Published:** 2024-04-26

**Authors:** Ting Wang, Mengjie He, Xudong Zhang, Zhixun Guo, Pinghan Wang, Fangyi Long

**Affiliations:** 1 Department of Clinical Research, Sichuan Clinical Research Center for Cancer, Sichuan Cancer Hospital and Institute, Sichuan Cancer Center, Affiliated Cancer Hospital of University of Electronic Science and Technology of China, Chengdu, 610041 China; 2 Laboratory Medicine Center, Sichuan Provincial Maternity and Child Health Care Hospital, Affiliated Women’s and Children’s Hospital of Chengdu Medical College, Chengdu Medical College, Chengdu, 610041 China

**Keywords:** Cancer, Circular RNAs (circRNAs), Autophagy, Therapeutic resistance

## Abstract

Cancer therapeutic resistance remains a significant challenge in the pursuit of effective treatment strategies. Circular RNAs (circRNAs), a class of non-coding RNAs, have recently emerged as key regulators of various biological processes, including cancer progression and drug resistance. This review highlights the emerging role of circRNAs-mediated autophagy in cancer therapeutic resistance, a cellular process that plays a dual role in cancer by promoting both cell survival and death. Increasing evidence suggests that circRNAs can modulate autophagy pathways, thereby influencing the response of cancer cells to therapeutic agents. In this context, the intricate interplay between circRNAs, autophagy, and therapeutic resistance is explored. Various mechanisms are discussed through which circRNAs can impact autophagy, including direct interactions with autophagy-related genes, modulation of signaling pathways, and cross-talk with other non-coding RNAs. Furthermore, the review delves into specific examples of how circRNA-mediated autophagy regulation can contribute to resistance against chemotherapy and radiotherapy. Understanding these intricate molecular interactions provides valuable insights into potential strategies for overcoming therapeutic resistance in cancer. Exploiting circRNAs as therapeutic targets or utilizing them as diagnostic and predictive biomarkers opens new avenues for developing personalized treatment approaches. In summary, this review underscores the importance of circRNA-mediated autophagy in cancer therapeutic resistance and proposes future directions for research in this exciting and rapidly evolving field.

## Introduction

Cancer continues to be a significant global health concern, with its incidence and mortality rates escalating despite advances in therapeutic approaches [1]. One of the foremost challenges in cancer treatment is the development of resistance to various therapeutic interventions, including chemotherapy, radiotherapy, hormone therapy, targeted therapy, and immunotherapy [2]. Therapeutic resistance often leads to treatment failure, disease progression, and decreased patient survival rates [3–5]. Cancer therapeutic resistance are associated with various factors, including autophagy, apoptosis, non-coding RNAs regulation, glutathione metabolism and ferroptosis [5–7]. Unraveling the molecular mechanisms underlying the therapeutic resistance has become a focal point of cancer research, driving the quest for innovative strategies to enhance treatment efficacy.

In recent years, non-coding RNAs (ncRNAs) have emerged as crucial regulators of diverse cellular processes, with circular RNAs (circRNAs) being recognized as a class of ncRNAs with intriguing regulatory roles [8]. CircRNAs are characterized by their covalently closed loop structure, rendering them resistant to RNA degradation pathways [9, 10]. This unique structural feature, along with their widespread presence across species and cell types, has garnered increasing attention toward understanding their functional relevance in health and disease [4, 11]. Of particular interest is the intricate interplay between circRNAs and autophagy, a conserved cellular degradation process that plays a pivotal role in maintaining cellular homeostasis and responding to stress [12].

Autophagy has been identified as a key player in cancer therapeutic resistance. By enabling cells to survive and adapt under adverse conditions induced by therapeutic agents, autophagy serves as a double-edged sword, both promoting cell survival and potentially contributing to treatment resistance [13–18]. The modulation of autophagy pathways has thus emerged as an attractive target for circumventing therapeutic resistance and enhancing treatment outcomes. Recent studies have begun to unveil the intriguing connections between circRNAs and autophagy, suggesting a potential nexus between these two regulatory networks in the context of cancer therapeutic resistance. CircRNAs can modulate autophagy at various stages through different mechanisms, including sponging miRNAs to regulate autophagy-related genes and participating in signaling pathways involved in autophagy regulation. By elevating or inhibiting autophagy activity, circRNAs influence the response of cancer cells to chemotherapeutic and radiation treatments, thereby impacting therapy sensitivity and resistance. For instance, elevated level of *circCPM* was detected in gastric cancer (GC) patients with 5-FU resistance. *CircCPM* functioned as a sponge to bind miR-21-3p, which indirectly augmented the expression of PRKAA2, leading to enhanced autophagic activity and resistance to 5-FU in GC [19]. Moreover, *circBANP* was found to be up-regulated in colorectal cancer (CRC), which reduced the level of miR-338-3p, thereby inducing autophagy and contributing to X-ray resistance in CRC [20]. However, the precise mechanisms of circRNA action, such as their potential role in autophagy-related protein recruitment or translation, remain largely unknown. Further study is needed to fully map circRNA functions, and investigating the roles of circRNAs in mediating autophagy in the context of cancer therapeutic resistance holds promise for deciphering new therapeutic targets and biomarkers.

This review aims to provide a comprehensive overview of the emerging role of circRNA-mediated autophagy in cancer therapeutic resistance. We summarize the intricate molecular mechanisms through which circRNAs influence autophagy pathways, discuss their potential clinical implications as diagnostic markers and therapeutic targets, and highlight the opportunities for exploiting this knowledge to develop novel strategies for overcoming cancer therapeutic resistance. Through a deeper understanding of the interplay between circRNAs and autophagy, this review seeks to contribute to the advancement of personalized and effective cancer treatment approaches.

## CircRNAs: structure, biogenesis, and functions

CircRNAs, a class of non-coding RNA molecules, possess a unique circular structure forming a closed loop structure where the 3ʹ and 5ʹ ends are covalently linked. This circular structure, resulting from a back-splicing event, makes circRNA resistant to degradation by exonucleases ribonuclease R (RNase R), giving them increased stability compared to linear RNAs [4]. This stability enabling circRNAs to persist, accumulate, and influence cellular regulation over an extended period, potentially contributing to the dysregulation of critical signaling pathways in cancer therapeutic resistance. Furthermore, the stability of circRNAs may also impact their interactions with other molecules, such as miRNAs or proteins, which are known to be modulators of therapeutic response.

CircRNAs have diverse functions ranging from miRNA sponging and post-transcriptional regulation to potential protein encoding. Numerous studies have demonstrated the capacity of circRNAs to mediate different biological processes through several mechanisms. These mechanisms include the recruitment of proteins to specific locations [21], serving as coordinators of RNA binding proteins (RBPs) [22], initiating translation for novel proteins [23], regulating parental genes transcription [24], establishing scaffolds for enzyme–substrate interactions [25], and more broadly, acting as molecular decoys for miRNAs [19, 20]. CircRNAs contain multiple binding sites for specific miRNAs, allowing them to sequester these miRNAs and prevent them from targeting their intended mRNA transcripts. This sequestration prevents miRNA-mediated mRNA degradation or translational inhibition, effectively influencing the expression of various genes involved in pathways such as cell proliferation, differentiation, and apoptosis [9].

**Fig. 1:**
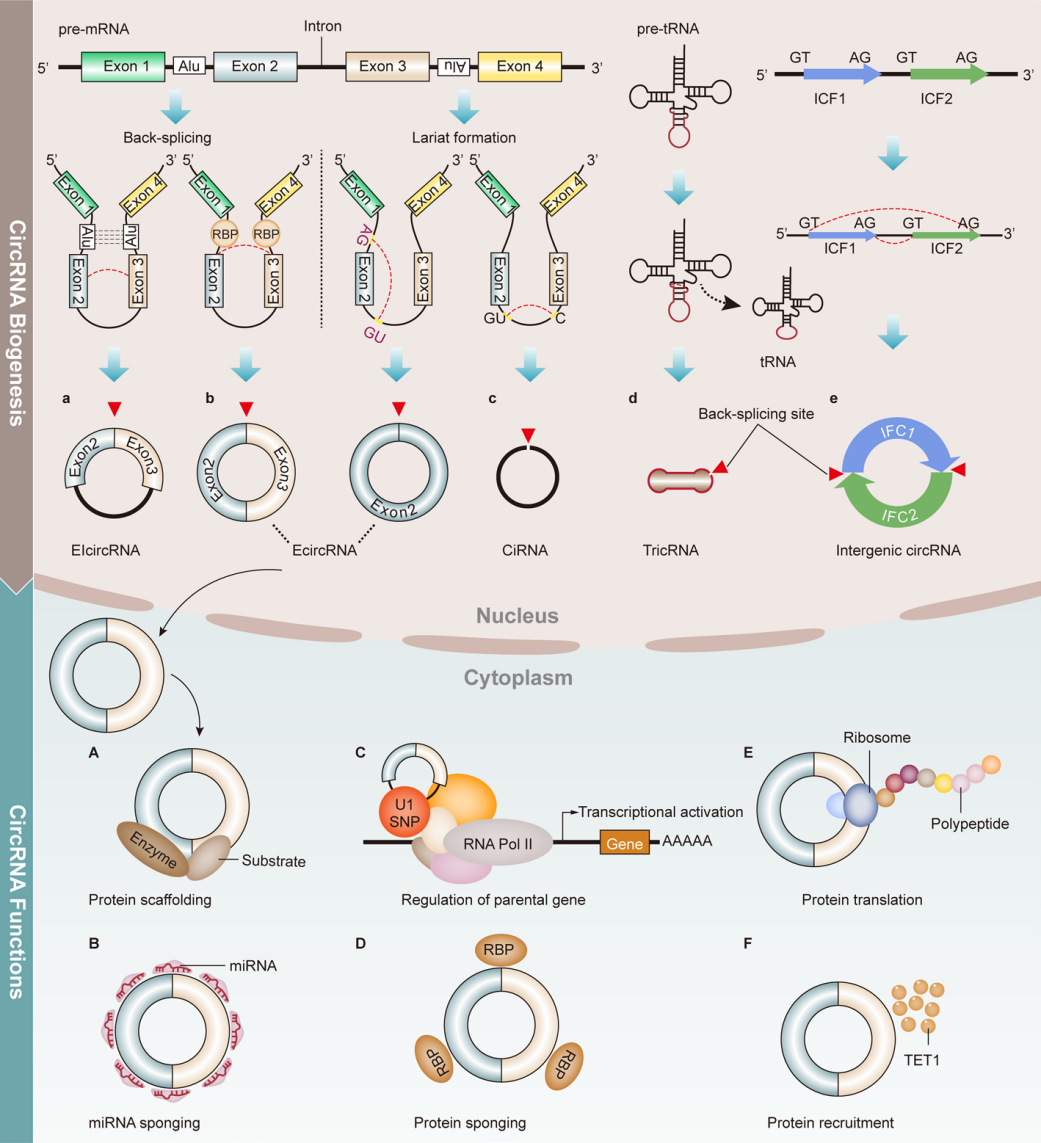
Overview of the autophagy process and regulatory mechanism. Autophagy consists of five main steps: initiation, phagophore nucleation, vesicle elongation, docking, and fusion and degradation. Autophagy is triggered by various stresses, such as hypoxia, oxidative stress, or energy nutrient deprivation. Under these conditions, the AMPK signaling is activated, which results in the dissociation of the ULK1/2 complex (comprising ULK1/2, ATG101, FIP200 and ATG13) from the mTORC1 complex (including GβL, mTOR, and Raptor), thereby promoting phagophore nucleation by the phosphorylating PI3KC3 complex (comprising Beclin1, AMBRA1, VPS15, VPS34, and ATG14L), and the production of local PtdIns3P in the omegasome. PtdIns3P then recruits PtdIns3P-binding proteins, such as WIPIs, DFCP1 and ATG9 vesicles, for phagophore expansion. Two ubiquitin-like conjugation systems are involved in vesicle elongation and autophagasome formation. In one system, the ATG12-ATG5-ATG16L complex (E3) is formed under the catalysis of ATG7 (E1-like enzyme) and ATG10 (E2-like enzyme). In the other system, cytosolic LC3-I is conjugated to phosphatidylethanolamine (PE) under the catalysis of protease ATG4, ATG3 and ATG7 to form membrane-bound LC3-PE complex (LC-II). Finally, a large group of molecules, including SNARE complex, HOPS complex, RAB GTPases and adaptors, cytoskeleton components, and related motor proteins, promote the fusion of the autophagosome and lysosome to form autolysosome, where the inner autophagosomal membrane and sequestered contents are degraded by lysosomal hydrolases and released for reutilization

Categorized based on their origins and formation mechanisms, circRNAs play diverse roles crucial for understanding cancer and therapeutic resistance. The main types include: (1) Exon–intron circRNAs (EIcircRNAs), formed through back-splicing, comprising both exonic and intronic sequences; (2) Exonic circRNAs (EcircRNAs), derived exclusively from gene exons, are formed through back-splicing of exons during pre-mRNA splicing; (3) Intronic circRNAs (CiRNAs), originating from gene introns, formed through lariat-driven circularization; (4) Intergenic circRNAs, generated by circularization of intron-containing fragments (ICFs) with GT-AG splicing sites, such as tRNA intronic circRNAs (TricRNAs) [4, 11, 26]. Following their formation, circRNAs exhibit diverse functions depending on their subtypes and cellular localization. For instance, exonic circRNAs, mainly in the cytoplasm, act as miRNA sponges and interact with RNA-binding proteins, influencing processes linked to cancer. In contrast, intronic circRNAs, primarily in the nucleus, are thought to play roles in gene regulation, including transcription and alternative splicing (as depicted in Fig. 1). Altered expression of circRNAs has been observed in different types of cancer, with specific circRNAs associated with cancer-related processes such as cell proliferation, invasion, and metastasis. CircRNAs are abundantly expressed in human tissues and body fluids, such as the brain, urine, blood, and saliva, and some have been identified as potential biomarkers for cancer diagnosis and prognosis [27]. The classification and functional diversity of circRNAs underscore their significance in cancer biology and therapeutic resistance.

## Autophagy and cancer therapeutic resistance

### Overview of autophagy

Autophagy is a fundamental cellular process that plays a critical role in maintaining cellular homeostasis by recycling and degrading damaged or obsolete cellular components. It is a highly regulated and dynamic process that involves the formation of a double-membrane vesicles called phagophore, which sequester targeted cellular components such as proteins or organelles to form autophagosome. The autophagosome then fuses with lysosomes for degradation.

**Fig. 2:**
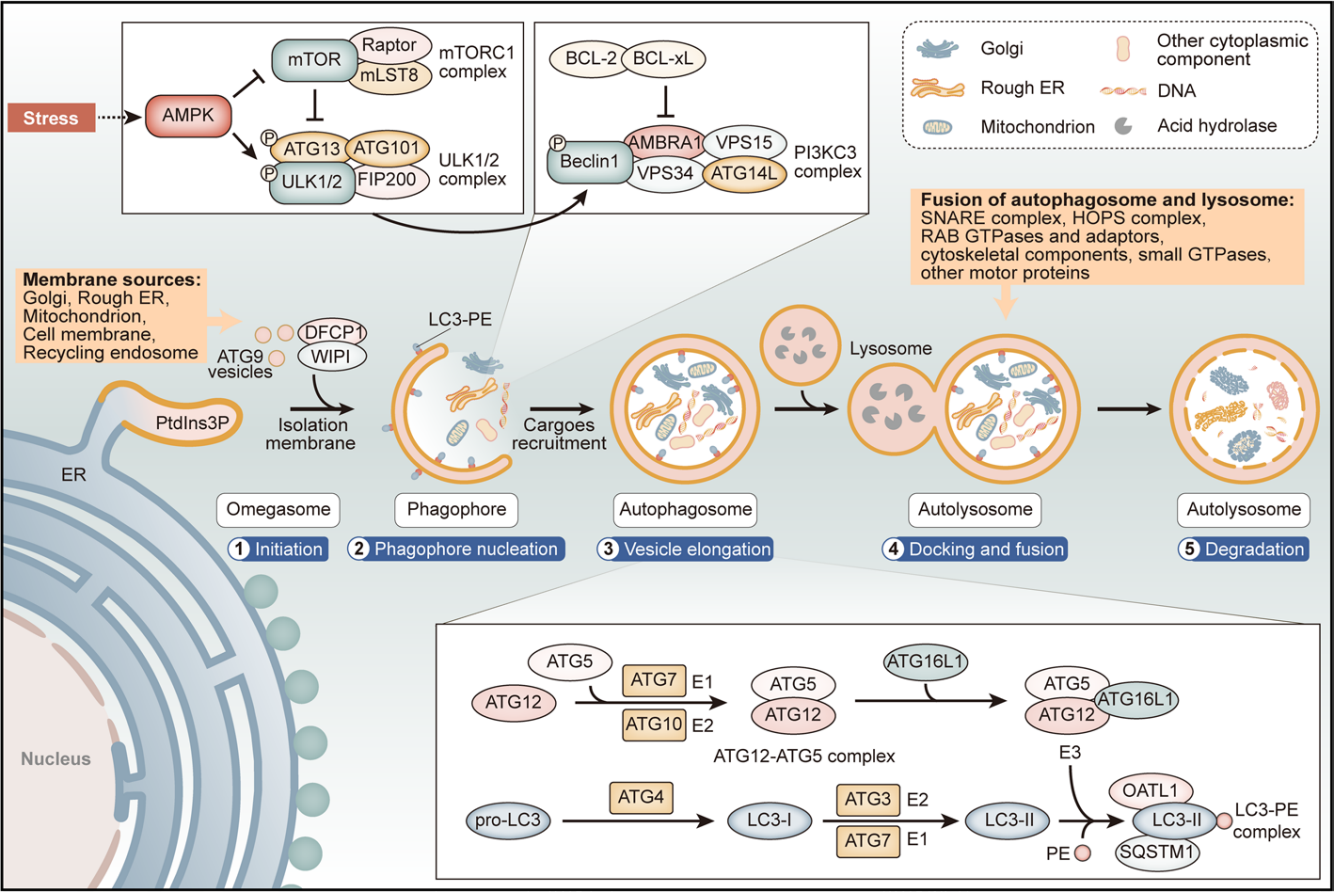
The mechanisms by which autophagy modulation affects chemosensitivity are multifaceted and involve multiple signaling pathways. The PI3K/Akt/mTOR pathway suppresses autophagy, while PTEN activation, as well as CaMKKβ, ATM or LKB1-mediated AMPK activation, can promote autophagy by activating the ULK1/2 complex or deactivating mTOR. Autophagy is also induced by Src/STAT3 signaling pathway through HO-1 activation and the ERK/JNK signaling pathway activated by HMGB1. Additionally, ROS stimulates autophagy by inhibiting JAK2/STAT3 and p38/mTOR pathways, while the IL-6/STAT3 pathway activates ERK and induces autophagy by repressing ERS. These signaling pathways further regulate the sensitivity of tumor cells to chemotherapy through the activation or hyperactivation of autophagy

Autophagy comprises a series of well-coordinated steps that culminate in the degradation of cellular components [28–34]. These steps can be summarized as follows (Fig. 2): (1) Initiation: autophagy is initiated by the activation of a protein complex called the ULK1/2 (Unc-51 like autophagy activating kinase 1/2) complex, which includes ULK1/2, ATG13, ATG101, and FIP200. This complex senses nutrient and energy status and regulates autophagosome formation. [32–34]. (2) Phagophore nucleation: this step involves the formation of a phagophore, an isolation membrane that eventually becomes the autophagosome. The process requires the activation of the class III phosphatidylinositol 3-kinase complex (PI3KC3), which includes Beclin-1 and VPS34 [10, 35]. The PI3KC3 complex catalyzes the formation of phosphatidylinositol 3-phosphate (PtdIns3P), a critical step contributing to phagophore nucleation [36, 37]. (3) Vesicle elongation: autophagosome membrane elongation and closure is facilitated by the conjugation of ATG5-ATG12 and LC3 (microtubule-associated protein 1A/1B-light chain 3) proteins [10, 38, 39]. LC3 is cleaved and lipidated to form LC3-II, which is essential for autophagosome formation [39, 40]. Autophagy receptors, such as p62/SQSTM1, recognize and bind to cargo destined for degradation, targeting it to autophagosomes [41, 42]. (4) Docking, fusion, and (5) degradation: as the autophagosome matures, it eventually fuses with a lysosome, forming an autolysosome. Within the autolysosome, lysosomal proteases degrade the autophagic cargo, which then becomes available for cellular reuse [28].

### Autophagy in chemoresistance

**Fig. 3:**
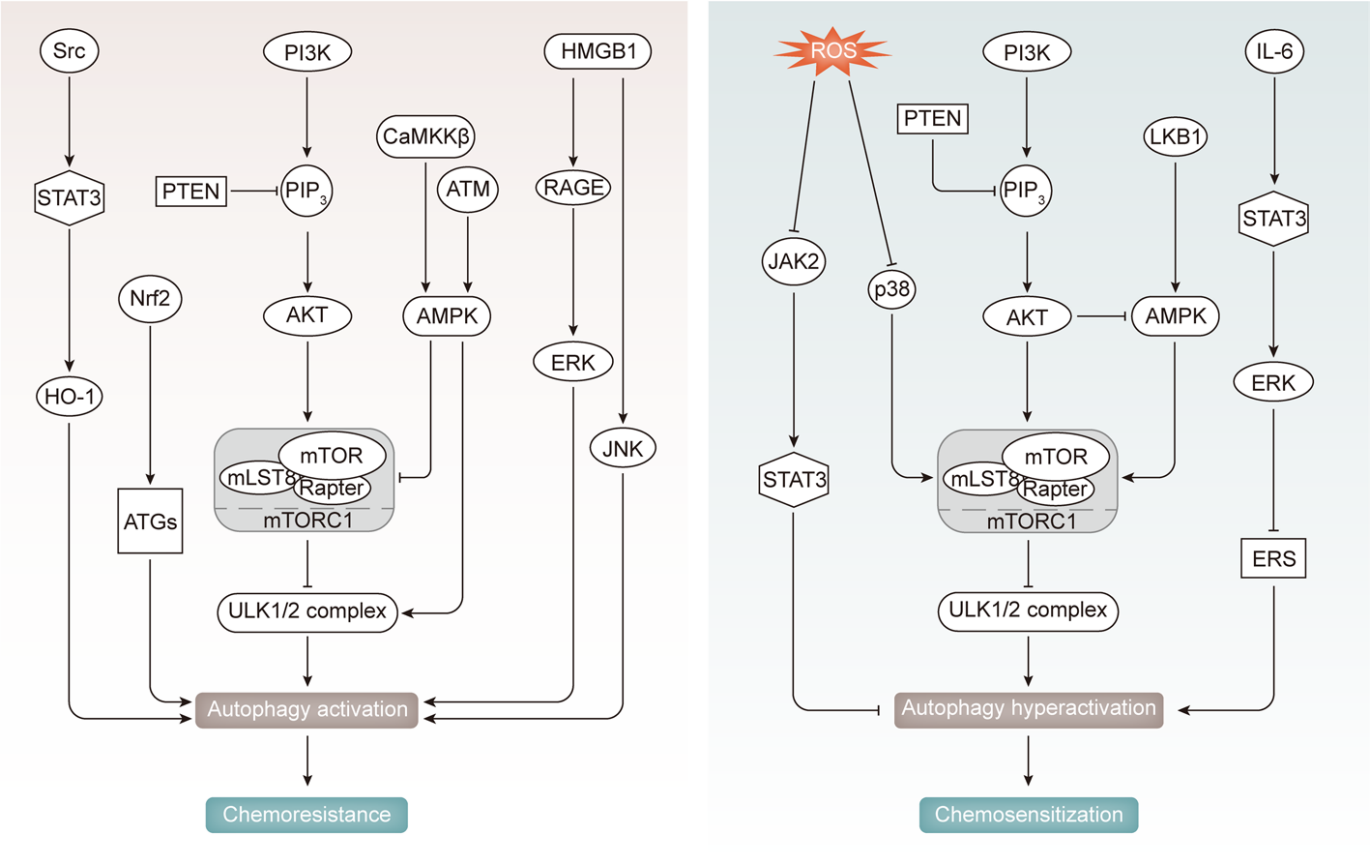
The regulation of autophagy to affect radiosensitivity involves a complex network of signaling pathway. Ionizing radiation (IR) and c-Jun inhibition activate the PI3K/Akt/mTOR pathway and inhibit autophagy, while PTEN, LKB1, and MEK/ERK mediated AMPK activation induces autophagy by blocking PI3K and mTOR activation. IR inhibits Wnt signaling and enhances autophagy through p62 disinhibition. ROS mediated signaling pathways (such as Nrf2/CaMKIIα, ERS, and NF-κB) can either facilitate or repress autophagy. IR-induced DNA damage triggers autophagy via the activation of ATM-related pathways. The modulation of autophagy by these signaling pathways further contributes to the enhancement or attenuation of resistance in cancer radiotherapy

Autophagy plays a dual role in chemoresistance, exhibiting both pro-resistance and pro-sensitivity effects. On one hand, autophagy can promote cancer cell survival under stress, enhancing resistance to chemotherapy. Inhibition of autophagy can sensitize cancer cells to anti-cancer agents. Conversely, excessive autophagy can induce cytotoxicity, augmenting chemotherapy effectiveness against drug-resistant tumors [11]. This duality makes autophagy a double-edged sword in cancer chemoresistance (as depicted in Fig. 3), modulated by various signaling pathways discussed below.

The PI3K/AKT/mTOR pathway negatively regulates autophagy by inhibiting the ULK1/2 complex [43, 44]. PTEN suppresses PI3K, while mTOR downstream effector P70S6K inactivates autophagy [43, 44]. Dysregulation of this pathway affects drug efficacy. Inhibition of PI3K/AKT/mTOR induces cell apoptosis and enhances doxorubicin (DOX) efficacy in breast cancer (BC) [45]. Conversely, PTEN activation contributes to autophagy-mediated DOX chemoresistance in BC [46]. Downregulation of AKT/mTOR promotes cisplatin (DDP) resistance in cervical cancer (CC) [47].

AMPK, an energy sensor, stimulates autophagy by phosphorylating ULK1/2 and inhibiting mTORC1 through Raptor phosphorylation [48]. AMPK impacts cancer cell chemosensitivity. Upregulated ATM and AMPK/ULK1 pathway induce autophagy, reducing temozolomide (TMZ) cytotoxicity in glioma [49]. CaMKKβ-mediated AMPKα/mTOR activation promotes autophagy, enhancing adriamycin (ADR) resistance in BC [50]. Intensified autophagy via LKB1/AMPK reverses docetaxel (DTX) resistance in prostate cancer (PC) [51]. In TMZ-resistant glioblastoma multiforme (GBM), autophagy sensitizes TMZ treatment by inhibiting the AKT/AMPK/mTOR pathway [52].

STAT3 signaling regulates autophagy bidirectionally depending on cellular location [53]. Dysfunctional autophagy via STAT3 signaling affects drug efficacy. Src/STAT3/HO-1 pathway stimulation in BC promotes cytoprotective autophagy, reducing DOX efficacy [54]. Conversely, ROS-mediated repression of JAK2/STAT3 activates autophagy, decreasing DDP tolerance in ovarian carcinoma (OC) [55]. Activated IL-6/STAT3/ERK pathways impair autophagy, leading to DDP resistance in OC [56]. Various signals like HMGB1/JNK, HMGB1/RAGE/ERK, and Nrf2/ATGs activate pro-survival autophagy, causing drug resistance [57–59]. Activation of p38/mTOR inhibits pro-death autophagy, inducing DDP tolerance in tongue squamous cell carcinoma [60].

### Autophagy in cancer radioresistance

**Fig. 4:**
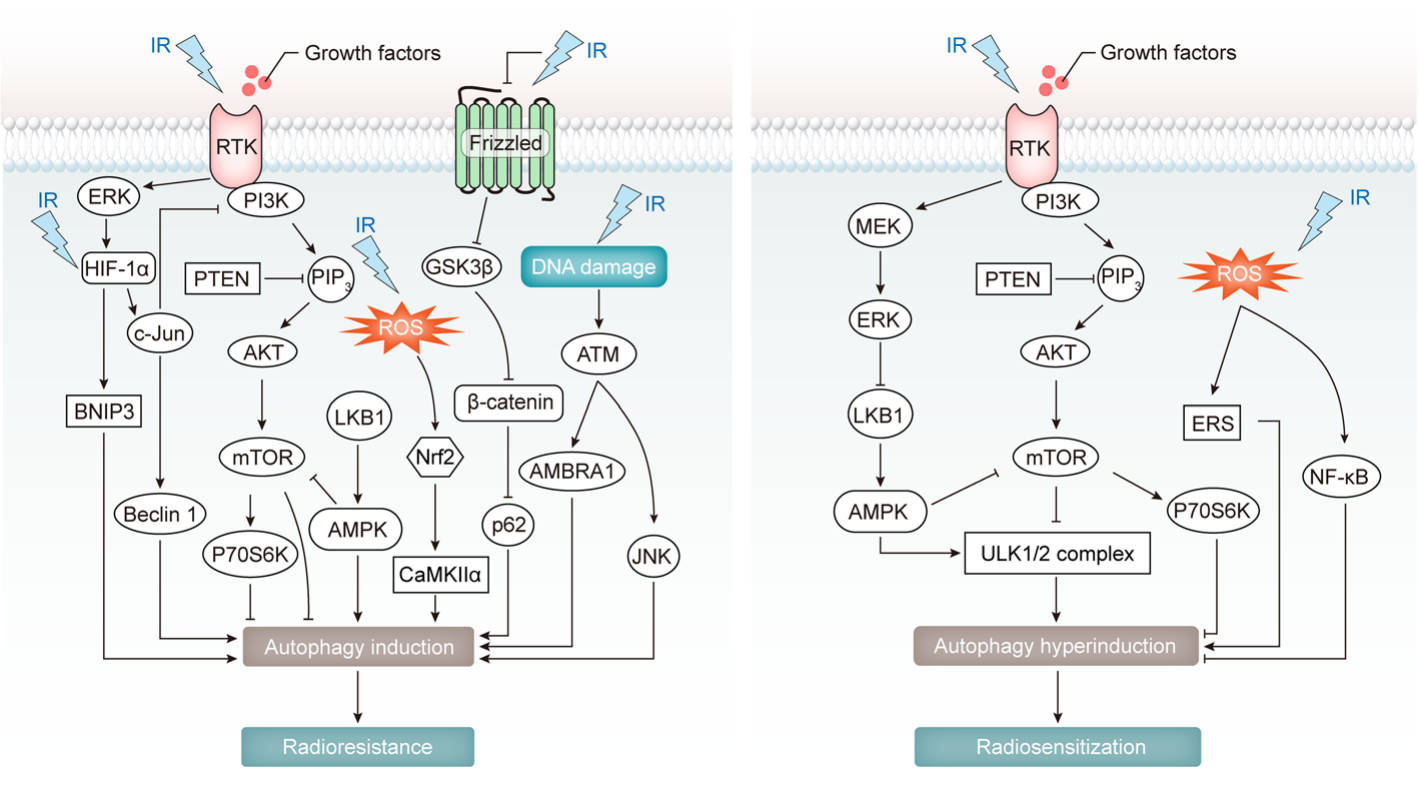
Biogenesis and functions of circRNAs. (1) CircRNAs are primarily generated by back-splicing of pre-mRNA in the nucleus. Long flanking introns complementary sequences (such as inverted repeat elements *Alu* pairs) and *trans acting* RNA binding proteins (RBPs) participate in back-splicing to form the circular structures. CircRNAs can also be produced by lariat-intron intermediate. The cyclization of lariat is driven by GU-rich element at the upstream splicing site and C-rich element near the downstream branching point, or AG-GU motifs at both ends. In addition, circRNAs can be generated during the process of pre-tRNA sequence splicing to maturation. Furthermore, a new class of circRNAs can be formed by the cyclization of two intron-containing fragments (ICFs), with GT-AG splicing signals on both sides. (2) There are five types of circRNAs, including (a) EIcircRNA, (b) EcircRNA, (c) CiRNA, (d) TricRNAs and (e) Intergenic circRNAs. (3) It has been reported that circRNAs possess a variety of regulatory functions, such as (A) protein scaffolding, (B) miRNA sponging, (C) regulation of parental gene, (D) protein sponging, (E) protein translation and (F) protein recruitment

Autophagy has a complex role in the response of cancer cells to radiation-induced stress, similar to its role in chemoresistance. While most studies suggest that autophagy induction can increase radioresistance, some experiments indicate that enhancing autophagy activity can restore radiosensitization. Various signaling pathways modulated by ionizing radiation (IR) can further influence therapy efficacy by regulating autophagy (Fig. 4).

The PI3K/AKT/mTOR pathway plays a crucial role in IR-mediated autophagy regulation [61–63]. Activation of this pathway, when inhibited by c-Jun, restrains protective autophagy, enhancing X-ray-induced cytotoxicity in nasopharynx cancer (NPC) [61]. Conversely, mTOR activation can facilitate autophagic cell death, increasing γ-ray cytotoxicity in EGFR mutant non-small cell lung cancer (NSCLC) [62]. Inactivation of the AKT/p70S6K pathway releases autophagy repression, promoting γ-radiation sensitivity in malignant glioma cells [63].

HIF-1α signaling, induced by IR-related hypoxia stress, influences autophagy and contributes to cancer radioresistance [64, 65]. For instance, HIF-1α activates c-Jun/Beclin1 signaling in lung cancer cells, initiating pro-survival autophagy and enhancing radioresistance [64]. LMP1 activates ERK/HIF-1α signaling, indirectly triggering autophagy and rendering nasopharynx cancer (NPC) cells insensitive to X-rays [65].

The Wnt-autophagy axis and the AMPK pathway also affect cancer IR therapy sensitivity [66–70]. Fractionated IR activates the Wnt/β-catenin/GSK3β/P62 pathway, promoting autophagic activity and enhancing radioresistance in glioblastoma (GBM) [67]. Upregulation of the LKB1/AMPK pathway by IR induces cytoprotective autophagy, contributing to radioresistance in esophageal squamous cell carcinoma (ESCC) [69, 70].

Several other signaling pathways, including Nrf2/CaMKIIα, ATM/JNK/AMBRA1, ROS/ERS, NF-κB, and MEK/ERK, also influence radiotherapy sensitivity by modulating autophagy activity [71–75]. In summary, autophagy's role in cancer radioresistance is multifaceted, with context-dependent effects. Understanding how different signaling pathways intersect with autophagy in response to radiation can provide insights into strategies for enhancing the effectiveness of radiation therapy.

## CircRNAs-mediated therapeutic resistance

**Fig. 5:**
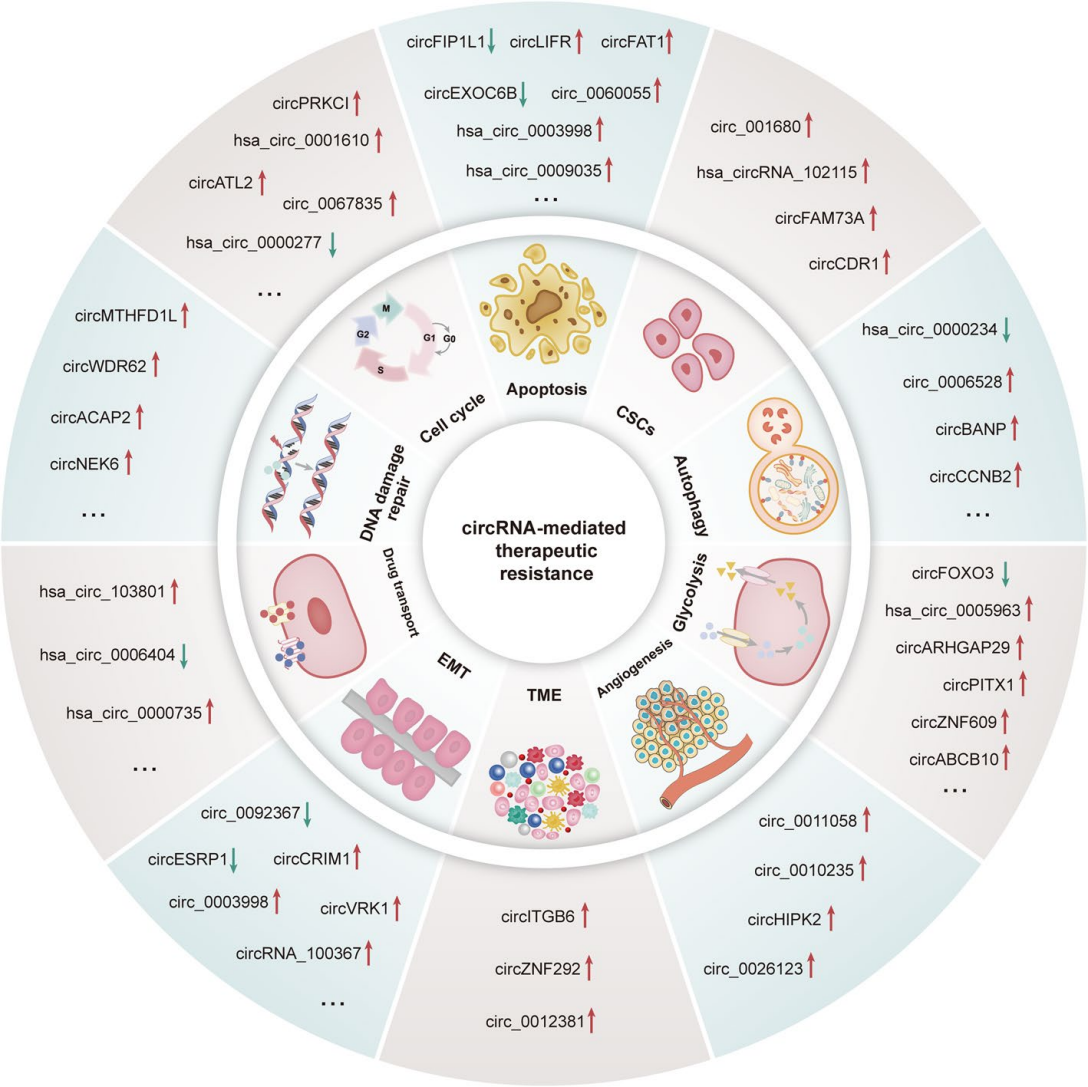
Aberrant expression of circRNAs is associated with cancer therapy resistance. Dysregulated circRNAs can regulate drug transporters (e.g., *hsa_circ_103801* and *hsa_circ_0000735*), EMT (e.g., *circESRP1* and *circVRK1*), TME (e.g., *circITGB6* and *circ_0012381*), angiogenesis (e.g., *circ_0011058* and *circHIPK2*), glycolysis (e.g., *circFOXO3* and *circPITX1*), autophagy (e.g., *hsa_circ_0000234* and *circBANP*), CSCs (e.g., *circCDR1* and *hsa_circRNA_102115*), apoptosis (e.g., *circLIFR* and *circFIP1L1*), cell cycle arrest (e.g., *circATL2* and *circ_0067835*), and DNA damage repair (e.g., *circMTHFD1L* and *circACAP2*). Upward-pointing red arrow ‘↑’ indicates upregulation, and downward-pointing green arrow ‘↓’ indicates downregulation

The above content suggests that autophagy is a key cellular event, which not only participates in normal biological processes but also plays an important role in the treatment response of cancer cells. In recent years, studies have shown that circRNA can regulate different stages of autophagy through various mechanisms, thereby affecting the response of cancer cells to therapy. In addition to regulating autophagy, circRNA can also influence cancer cell treatment resistance through several other mechanisms. For example, circRNAs can regulate the expression levels of drug transport proteins, thereby altering the accumulation of drugs within cells [76, 77]. Additionally, circRNAs can regulate the apoptosis process of cancer cells [78–81], affect the degree of epithelial-mesenchymal transition in cancer cells [82–85], modulate glycolytic metabolism [86–88], regulate factors related to cancer stem cell properties [89–91], participate in tumor angiogenesis [92–94], regulate cell cycle progression [95, 96], influence DNA damage repair [97–100], and regulate the tumor microenvironment [101]. In summary, circRNA plays an important role in cancer therapeutic resistance through multiple mechanisms, as depicted in Fig. 5.

### Regulation of drug transporters

Drug efflux transporters such as P-glycoprotein (P-gp), breast cancer resistance protein (BCRP), and multidrug resistance proteins (MRPs) play an important role in the development of therapeutic resistance in cancer. By pumping drugs out of cancer cells, these transporters decrease the intracellular accumulation of chemotherapy agents, reducing their efficacy. Certain circRNAs have been shown to regulate the expression of these efflux transporters, thereby modulating drug resistance. For instance, *hsa_circ_103801* has been shown to increase cisplatin resistance in osteosarcoma cells by regulating MRP1 and P-gp [76]. Addtionally, *hsa_circ_0006404* and *hsa_circ_0000735* modulate docetaxel resistance in ovarian cancer cells through P-gp regulation [77]. Upregulation of transporters mediated by circRNAs can elevate drug efflux and contribute to resistance to chemotherapy treatments.

### Influence on apoptosis process

In addition to regulating drug transporters, circRNAs can also affect therapeutic resistance by modulating apoptosis, a key process in determining treatment responses. Apoptosis serves both pro-survival and pro-death roles in cancer. Some studies have shown circRNAs can influence apoptosis and thereby therapeutic sensitivity through different mechamisims. For example, *circLIFR* and *circEXOC6B* were found to enhance apoptosis in drug-resistant bladder and ovarian cancer cell lines respectively, by forming RNA–protein binary complex with MSH2 and targeting miR-376c-3p [78, 79], which increased the cells’ vulnerability to therapy. In contrast, other circRNAs like *circFAT1* promote resistance to therapy by dampening apoptosis. Research found *circFAT1* increased oxaliplatin resistance in breast cancer by sponging miR-525-5p and inhibiting apoptosis [80]. Similarly, *hsa_circ_0003998* was shown to foster chemoresistance in lung adenocarcinoma by upregulating anti-apoptotic genes through a ceRNA mechanism [81]. In summary, circRNAs play divergent roles in modulating apoptosis, thus influencing cancer treatment responses.

### Influence on epithelial-mesenchymal transition (EMT)

EMT is a process where epithelial cancer cells lose their cell–cell adhesion and gain migratory and invasive properties associated with mesenchymal cells. EMT promotes cancer progression by enhancing cancer cell infiltration, migration and stem-like phenotypes, thus conferring therapeutic resistance [82]. Studies have shown that circRNAs can modulate the EMT process, and thereby impact treatment sensitivity. For example, *circ_0092367* and *circESRP1* inhibited EMT and increased chemosensitivity by regulating miR-1206 and miR-93-5p [82, 83]. However, *circCRIM1* and *circ_0003998* were found to promote EMT and resist docetaxel and doxorubicin treatment by acting as ceRNAs [84, 85].

### Modulation of glycolytic metabolism

Cellular glycolysis is crucial for cancer progression and metabolism reprogramming. Glycolysis converts glucose into pyruvate to generate energy and biosynthetic intermediates to support rapid cell growth and proliferation [87]. Studies have demonstrated that *circFOXO3* promotes glycolysis and enhances cisplatin sensitivity in NSCLC cells by regulating the miR-543/Foxo3 axis [86]. Conversely, exosomal *hsa_circ_0005963* targets miR-122 to up-regulate PKM2 in CRC cells, thereby promoting glycolysis and conferring oxaliplatin resistance [87]. Additionally, in DTX-resistant PC cells, EIF4A3-mediated *circARHGAP29* overexpression heightens aerobic glycolysis, enhancing resistance [88].

### Regulating cancer stem cells (CSCs)

CSCs are a small subset of cells that have self-renewal and tumor initiating abilities. They are thought to be responsible for tumor initiation, metastasis and resistance to therapy. CircRNAs have been shown to influence CSC properties and the stem cell-like phenotype, which is associated with therapy resistance. *CircCDR1*, expressed in NSCLC cells with DDP resistance, promotes HOXA9 levels by competitively binding miR-641, thereby enhancing stem-like properties [89]. Similarly, *circ_001680* in CRC interacts with miR-340 to up-regulate BMI1 in NPC cells, inducing stemness and irinotecan tolerance [90]. Moreover, *circFAM73A* in GC regulates the miR-490-3p/HMGA2 axis, promoting stemness and DDP resistance [91].

### Involvement in angiogenesis

Angiogenesis is the process of formation of new blood vessels. Rapid proliferation of tumors depends on sufficient blood supply from new vessel formation. Increased angiogenesis is associated with advanced diseases, metastasis and poor prognosis [92]. Studies found circRNAs modulated angiogenesis and influenced resistance. *CircHIPK2* in NSCLC augments VEGFA and miR-1249-3p binding, thereby promoting angiogenesis and DDP resistance [92]. Conversely, *circ_0010235* in PTX-resistant NSCLC suppresses angiogenesis via the miR-512-5p/FAM83F axis [93]. Additionally, *circ_0026123* in DDP-resistant OC promotes RAB1A by suppressing miR-543, contributing to angiogenesis [94].

### Participation in cell cycle progression

Deregulated cell cycle progression allows uncontrolled growth of cancer cells. CircRNAs are involved in modulating the cell cycle, which influences therapy response. *CircATL2* in OC induces cell cycle arrest and initiates PTX resistance via the miR-506-3p/NFIB axis [95]. Conversely, knockdown of *hsa_circ_0000277* in ESCC increases the G0/G1 phase by regulating the miR‐873-5p/SOX4 axis, promoting DDP resistance [96].

### Impact on DNA damage repair (DDR)

DDR pathways are crucial for maintaining genomic stability. Elevated DDR is linked to resistance by allowing cancer cells to repair DNA damage caused by therapy [97]. CircRNAs were shown to regulate DDR genes and modulate resistance. *CircMTHFD1L* in pancreatic cancer up-regulates RPN6 by acting as an miR-615-3p sponge, promoting GEM resistance [97]. Furthermore, exosomal *circWDR62* from glioma cells induces MGMT expression through miR-132, thereby weakening TMZ efficacy [98].

### Role in tumor microenvironment (TME)

TME refers to cellular and non-cellular components that surround and influence tumor cells. It includes fibroblasts, immune cells, blood vessels, extracellular matrix etc. The complex network of tumor-stromal cell interactions within the TME contributes significantly to therapeutic resistance [101]. Studies illustrated that circRNAs shape the TME by regulating components like tumor-associated macrophages. *CircITGB6* in OC facilitates IGF2BP2-mediated FGF9 expression and polarization of tumor-associated macrophages towards the M2 phenotype, conferring DDP resistance [101].

### Regulation of autophagy process

Autophagy acts as a double-edged sword in cancer by promoting both cell survival and death [11]. Dysregulated autophagy confers therapeutic resistance in cancers. Certain circRNAs were found to modulate autophagy and influence resistance by regulating autophagy-related genes or signaling pathways, such as *hsa_circ_0000234* and *circ_0006528* can influence cancer chemoresistance by regulating the autophagy process, which will be discussed in Sect. "CircRNAs-mediated autophagy modulation and their impact on cancer therapeutic resistance".

In summary, circRNAs exhibit multifaceted roles in treatment resistance, intricately sculpting drug transport, apoptosis, EMT, glycolysis, CSCs, angiogenesis, cell cycle, DDR, TME, and autophagy. Their dichotomous nature underscores the complexity of therapeutic resistance mechanisms, presenting promising avenues for targeted interventions [76–119].

## CircRNAs-mediated autophagy modulation and their impact on cancer therapeutic resistance

**Fig. 6:**
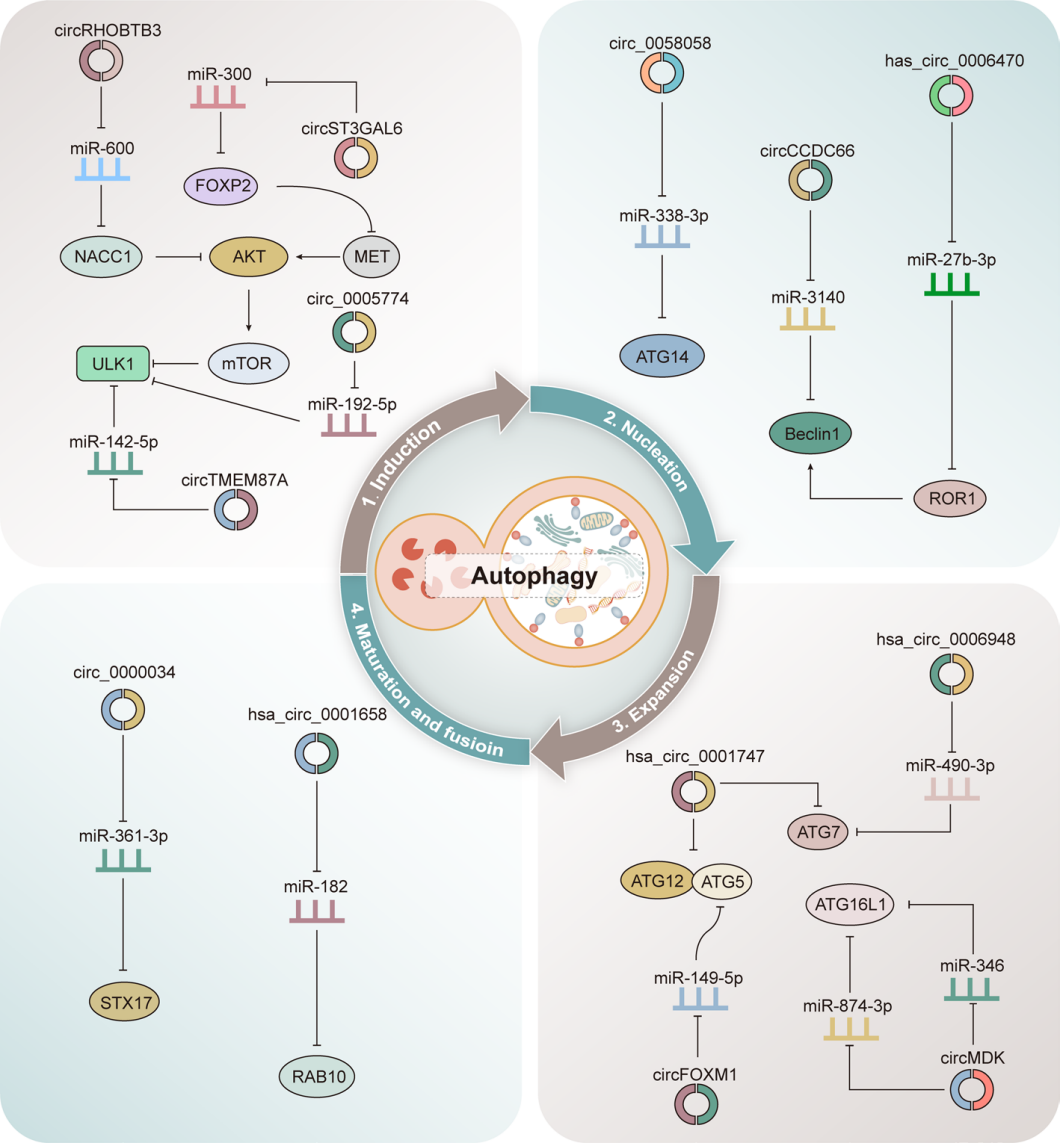
CircRNAs regulate autophagy and affect cancer development. The autophagy process can be modulated by circRNAs at various stages, leading to either suppression or promotion of cancer. This schematic diagram illustrates some representative circRNAs, including *circRHOBTB3*, *circCCDC66*, *circFOXM1*, *circ_0000034* and others, that impact cancer progression by regulating autophagy

CircRNAs can control various proteins involved in autophagy, a crucial process in tumor development. (1) In the initial stage of autophagy, circRNAs influence the PI3K/AKT/mTOR pathway, which regulates autophagy [43]. For instance, *circRHOBTB3* and *circST3GAL6* impact this pathway in pancreatic and gastric cancer cells, respectively, leading to pro-survival autophagy [120, 121]. *CircTMEM87A* and *circ_0005774* indirectly control autophagy by affecting proteins like ULK1, promoting autophagy and aiding cancer cell growth [122, 123]. (2) During the phagophore nucleation stage, certain circRNAs influence molecules like ATG14 and Beclin1, initiating autophagy. *Circ_0058058* and *circCCDC66* affect these molecules in multiple myeloma and colorectal cancer cells, respectively, promoting cancer cell behavior [124, 125]. *Hsa_circ_0006470* triggers autophagy by regulating Beclin1 in gastric cancer [126]. (3) In the membrane expansion phase, circRNAs impact the ATG5-ATG12-ATG16L complex and LC3 conjugation system, crucial for autophagosome elongation. CircRNAs like *hsa_circ_0001747*, *circMDK*, and *circFOXM1* influence these processes in various cancers, leading to autophagy activation and cancer progression [127–129]. *Hsa_circ_0006948* induces autophagy in osteosarcoma cells by affecting ATG7, promoting cancer development [130]. (4) In the late maturation and fusion stage, circRNAs indirectly control autophagy-related proteins like STX17 and RAB10. For instance, *circ_0000034* and *hsa_circ_0001658* impact retinoblastoma and gastric cancer cells, respectively, enhancing autophagy and cancer cell behavior [131, 132] (as shown in Fig. 6).

**Fig. 7:**
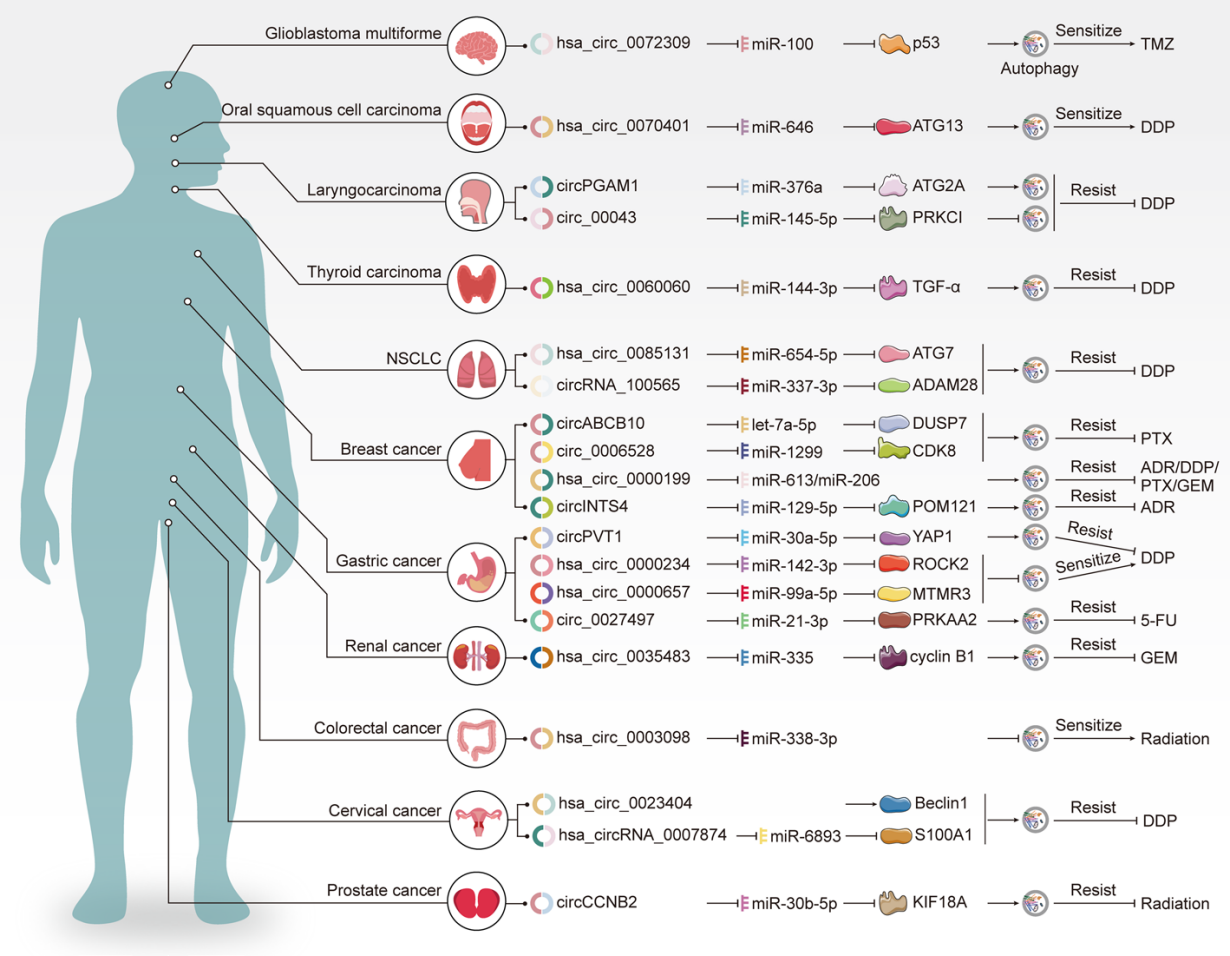
Dysregulated circRNAs are involved in autophagy-mediated resistance to chemotherapy and radiotherapy in cancer. The majority of circRNAs act as miRNA sponges, which sequester miRNAs and prevent them from regulating their downstream mRNA targets, thereby affecting the initiation of autophagy. Only a few circRNAs directly interact with proteins to modulate autophagy

Besides its crucial role in cancer development, autophagy assumes a dual role in therapy—it can either sensitize cancer cells to treatment-induced death or reforce their resistance, leading to therapeutic failure and disease relapse. This duality underscores the need for precise regulation of autophagy to enhance treatment efficacy. Notably, recent research has unearthed the regulatory prowess of non-coding RNAs, particularly circRNAs, in modulating autophagic pathways, influencing various stages of autophagy in diverse types of cancer (as illustrated in Fig. 7 and summarized in Table 1).

**Table 1:** The roles of autophagy associated-circRNAs in chemo- and radio-therapy of malignant tumors

Cancer types	CircRNAs	CircBase ID	Host gene	Sponge targets	Downstream genes/pathway	Impacts on autophagy	Functions	Refs.
GBM	circLIFR↓	hsa_circ_0072309	LIFR	miR-100	p53↑	Promotion	TMZ sensitivity	[137]
CRC	circBANP↑	hsa_circ_0003098	BANP	miR-338-3p	/	Promotion	Radioresistance	[20]
GC	circCPM↑	hsa_circ_0027497	CPM	miR-21-3p	PRKAA2↑	Promotion	5-FU resistance	[19]
	circCUL2↓	hsa_circ_0000234	CUL2	miR-142-3p	ROCK2↑	Inhibition	DDP sensitivity	[145]
	circPVT1↑	/	PVT1	miR-30a-5p	YAP1↑	Promotion	DDP resistance	[147]
	circMCTP2↓	hsa_circ_0000657	MCTP2	miR-99a-5p	MTMR3↑	Inhibition	DDP sensitivity	[149]
RC	circCCNB2↑	hsa_circ_0035483	CCNB2	miR-335	cyclin B1↑	Promotion	GEM resistance	[150]
TC	circEIF6↑	hsa_circ_0060060	EIF6	miR-144-3p	TGF-α↑	Promotion	DDP resistance	[154]
BC	circABCB10↑	hsa_circ_0008717	ABCB10	let-7a-5p	DUSP7↑	Promotion	PTX resistance	[155]
	circ_0006528↑	hsa_circ_0006528	PRELID2	miR-1299	CDK8↑	Promotion	PTX resistance	[156]
	circAKT3↑	hsa_circ_0000199	AKT3	miR-613/miR-206	PI3K/AKT/mTOR pathway↑	Inhibition	ADR/DDP/PTX/GEM resistance	[157]
	circINTS4↑	hsa_circ_0002476	INTS4	miR-129-5p	POM121↑	Promotion	ADR resistance	[158]
NSCLC	circPABPC1↑	hsa_circ_0085131	PABPC1	miR-654-5p	ATG7↑	Promotion	DDP resistance	[170]
	circRNA_100565↑	hsa_circ_0017956	MLLT10	miR-337-3p	DAM28↑	Promotion	DDP resistance	[172]
CC	circRNF121↑	hsa_circ_0023404	RNF121	miR-5047	/	Inhibition	DDP resistance	[176]
	circMTO1↑	hsa_circ_0007874	MTO1	miR-6893	/	Promotion	DDP resistance	[180]
Laryngocarcinoma	circPGAM1↑	/	PGAM1	miR-376a	ATG2A↑	Promotion	DDP resistance	[182]
	circPARD3↑	circ_00043	PARD3	miR-145-5p	PRKCI↑	Inhibition	DDP resistance	[184]
OSCC	circPKD2↓	hsa_circ_0070401	PKD2	miR‐204‐3p	ATG13↑	Promotion	DDP sensitivity	[186]
PC	circCCNB2↑	hsa_circ_0035483	CCNB2	miR-30b-5p	KIF18A↑	Promotion	Radioresistance	[191]

### Glioblastoma multiforme (GBM)

GBM, one of the most aggressive and malignant forms of brain cancer, poses a significant challenge in cancer treatment. TMZ is a standard chemotherapy drug used to treat GBM. However, resistance to TMZ often develops, leading to treatment failure. In this context, *hsa_circ_0072309* has emerged as a crucial regulator in GBM and other cancers [133–136]. Its down-regulation in GBM patients has been associated with poor prognosis. Notably, *hsa_circ_0072309* sensitizes GBM cells to TMZ by targeting miR-100/p53 axis and activating autophagy-induced apoptosis, providing a potential avenue to overcome TMZ resistance in GBM treatment [137].

### Colorectal cancer (CRC)

CRC is a prevalent malignancy with diverse molecular subtypes. Resistance to X-ray irradiation poses a significant challenge in CRC therapy. BANP protein, a human homolog of matrix attachment region (MAR) binding protein scaffold/matrix attachment region binding protein 1 (SMAR1), plays a crucial role in transcriptional suppression of multiple oncogenes [138]. *CircBANP (hsa_circ_0003098)* has been identified as a key player in CRC, particularly in radiation resistance [139, 140]. Its up-regulation in CRC patients resistant to X-ray irradiation highlights its role in promoting autophagy. Silencing *circBANP* has been found to modulate miR-338-3p/LC3B/p62 axis and deactivate autophagy, thereby enhancing the efficacy of X-ray-induced tumor suppression in CRC [20].

### Gastric cancer (GC)

Gastric Cancer (GC) is characterized by its aggressive nature and resistance to various chemotherapeutic agents. Resistance to drugs like 5-Fluorouracil (5-FU) is a common challenge in GC treatment. *CircCPM*, *CircCUL2*, *CircPVT1*, and *CircMCTP2* have emerged as key regulators influencing autophagy and chemoresistance in GC.

*CircCPM* is highly expressed in 5-FU-resistant GC patients, indicating poor survival outcomes. It acts as a sponge for miR-21-3p, enhancing the expression of AMPK subunit alpha 2 (PRKAA2) and promoting autophagy. This reduced apoptosis in GC cells, contributing to 5-FU tolerance [19]. In addition, dysregulation of its parental gene CPM has been implicated in many types of cancer [141].

*CircCUL2* (*hsa_circ_0000234*) acted as a tumor-suppressive and regulator to sensitize tumor cells to chemotherapeutic drugs [142–144], is down-regulated in GC patients' serum and tissues, and it is positively correlated with overall survival. When combined with miR-142-3p, it upregulate ROCK2, reducing autophagy in GC cells and increasing sensitivity to DDP [145].

CircPVT1, originating from the plasmacytoma variant translocation 1(PVT1) gene, is present in exosomes from DDP-resistant GC cells. It competitively binds with miR-30a-5p, increasing yes-associated protein 1 (YAP1) levels. This upregulates LC3B and P-gp, promoting autophagy, reducing apoptosis, and inhibiting DDP's efficacy [146, 147].

There are two types of CircMCTP2 with distinct functions. One type, *circMCTP2* (*hsa_circ_0000658*), facilitates bladder carcinoma progression by regulating the miR-498/murine double minute-2 (MDM2) axis [148]. The other type, *circMCTP2* (*hsa_circ_0000657*), found at low levels in DDP-resistant GC cells, inhibits miR-99a-5p, promoting myotubularin-related protein 3 (MTMR3) expression. This downregulates LC3-II, weakens autophagy, and enhances DDP sensitivity [149].

These circRNAs, through intricate mechanisms, influence autophagy levels, enabling GC cells to survive and resist chemotherapy. Understanding their roles offers potential avenues for therapeutic interventions in GC treatment.

### Renal cancer (RC)

RC is a malignancy originating in the kidneys and is a significant public health concern globally. Gemcitabine (GEM) is commonly employed in chemotherapy for RC. However, the development of resistance to GEM presents a major challenge in the treatment of this cancer. Elevated levels of *hsa_circ_0035483* have been observed in human RC tissues. In RC cells treated with GEM, *hsa_circ_0035483* induces the expression of cyclin B1 by suppressing miR-335. This, in turn, increases the levels of LC3B and inhibits caspase3 expression, thereby activating autophagy and promoting resistance to GEM [150].

### Thyroid carcinoma (TC)

TC arises from the thyroid gland and is a prevalent cancer type worldwide. Cisplatin (DDP) is commonly used in TC chemotherapy. However, resistance to DDP poses a significant challenge in the effective treatment of TC. *CircEIF6 (hsa_circ_0060060)*, a circRNA spliced from the eukaryotic initiation factor 6 (eIF6) gene, is up-regulated in DDP-treated TC cells. EIF6 plays a key role in the biosynthesis and function of ribosomes, and dysregulation of eIF6 is closely associated with the formation and development of cancers [151–153]. In DDP-treated TC cells, *circEIF6* suppresses miR-144-3p, leading to increased expression of transforming growth factor-alpha (TGF-α), elevated LC3B levels, promotion of autophagy, and consequent DDP insensitivity [154].

### Breast cancer (BC)

BC is a heterogeneous disease with diverse molecular subtypes. Taxanes, such as Paclitaxel (PTX), are widely used in BC chemotherapy. However, resistance to PTX significantly hampers treatment outcomes. *CircABCB10*, circ_0006528, *hsa_circ_0000199*, and *circINTS4* have been identified as critical circRNAs involved in autophagy regulation and chemoresistance in BC. Their roles in activating autophagy pathways shed light on the intricate mechanisms of drug resistance in BC, providing potential avenues for targeted therapies [155–158].

*CircABCB10* (*hsa_circ_0008717*) has been implicated as an oncogene and a potential diagnostic and therapeutic target in various cancers [159]. In PTX-resistant BC tissues and cells, up-regulated circABCB10, derived from its parental gene ABCB10, interacts with let-7a-5p. This interaction increases the level of dual specificity phosphatase 7 (DUSP7), enhances LC3B-mediated autophagy, and leads to PTX resistance [155].

*Circ_0006528* is involved in BC progression and ADR tolerance through distinct mechanisms [160–162]. In PTX-intolerant BC patients, elevated *circ_0006528* interacts with miR-1299, promoting CDK8 enrichment, indirectly increasing P-gp and Beclin1 expression. This activation of autophagy induces PTX resistance in BC cell lines [156].

*Hsa_circ_0000199*, derived from the AKT3 gene, confers resistance to various chemicals, including ADR, DDP, PTX, and GEM in triple-negative breast cancer (TNBC) patients [157, 163]. It interacts with miR-613/miR-206, activating the PI3K/AKT/mTOR signaling pathway, dampening Beclin1 and LC3-II expression, attenuating autophagy, and enhancing chemical tolerance.

*CircINTS4* (*hsa_circ_0002476*) accelerates bladder cancer cell malignancy through the miR-146b/CARMA3 axis modulation [164]. Additionally, *circINTS4* overexpression was detected in TNBC patients with ADR resistance. Acting as a molecular sponge, *circINTS4* sequesters miR-129-5p, increasing POM121 expression. This indirect up-regulation of P-gp and Beclin1 triggers autophagy, inducing ADR tolerance [158].

### Non-small cell lung cancer (NSCLC)

NSCLC is the most common type of lung cancer. Platinum-based drugs, such as DDP, are frequently used in NSCLC chemotherapy. However, resistance to DDP poses a significant challenge in NSCLC treatment. *Hsa_circ_0085131* and *circRNA_100565* have been identified as key circRNAs in DDP resistance in NSCLC. Their roles in inducing autophagy shed light on the molecular mechanisms underlying DDP resistance, offering potential targets for overcoming resistance in NSCLC therapy [165–167].

*Hsa_circ_0085131*, derived from the polyadenylate-binding protein cytoplasmic 1 (PABPC1) gene transcript, is up-regulated in recurrent NSCLC patients [165, 169–172]. It acts as a sponge for miR-654-5p, regulating ATG7 and subsequent LC3-II expression [165]. This leads to autophagy induction, inhibits cell apoptosis, and ultimately contributes to DDP resistance.

*CircRNA_100565* (*hsa_circ_00179561*), derived from back-splicing of the 16th to 20th exons of the MLLT10 gene, increases the expression of high mobility group AT-hook 2 (HMGA2) by inhibiting miR-506-3p. This exacerbates the malignant behavior of NSCLC cells [166]. In DDP-resistant NSCLC patients, *circRNA_100565* is highly expressed, leading to elevated ADAM metallopeptidase domain 28 (ADAM28) levels through interaction with miR-337-3p. ADAM28 reinforces LC3B and Beclin1 levels, enhancing autophagy, suppressing apoptosis, and promoting cell proliferation and DDP insensitivity in NSCLC cells [167].

### Cervical cancer (CC)

CC originates in the cells lining the cervix. Resistance to DDP is a common issue in CC treatment. *Hsa_circ_0023404* and *circMTO1* have been identified as crucial players in DDP resistance in CC. Their roles in modulating autophagy provide valuable insights into the mechanisms underlying chemoresistance in CC, paving the way for targeted interventions [173–177].

*Hsa_circ_0023404*, originating from ring finger protein 121 (RNF121), acts as an oncogene in various cancers, including CRC, CC, and NSCLC [173, 178, 179]. Guo et al. demonstrated that increased *hsa_circ_0023404* expression in CC cells reduced Beclin1, increased p62 via miR-5047 sponge effect. This inhibition of autophagy decreased apoptosis and aggravated DDP resistance [174]. Additionally, *hsa_circ_0023404* reduced NSCLC sensitivity to DDP by activating the miR-646/SOX4 axis [180].

*CircMTO1* (*hsa_circ_0007874*) is derived from circularized exons 2–3 of mitochondrial tRNA translation optimization 1 (MTO1) gene and plays a dual role as a carcinogenic driver or tumor suppressor in cancer [175, 176]. Its up-regulation in CC patients indirectly triggers autophagy by sponging miR-6893, inhibiting apoptosis, and leading to DDP resistance [177].

### Laryngocarcinoma

Laryngocarcinoma, also known as laryngeal cancer, originates in the larynx. Resistance to DDP is also a significant concern in laryngocarcinoma treatment. *CircPGAM1* and *circPARD3* have been identified as key circRNAs influencing autophagy and DDP resistance in laryngocarcinoma. Understanding their roles in autophagy regulation provides crucial information for developing targeted therapies to overcome DDP resistance in laryngocarcinoma patients [181–183].

*CircPGAM1*, derived from phosphoglycerate mutase 1 (PGAM1) genome DNA, drives the malignant progression of epithelial OC cells via the miR-542-3p/CDC5L/PEAK1 pathway [184]. In laryngocarcinoma patients, increased *circPGAM1* expression correlates with advanced clinical stages and reduced overall survival rates. Acting as a miRNA sponge, *circPGAM1* targets miR-376a, enhancing ATG2A expression in laryngocarcinoma cells. This promotes autophagy, inhibits apoptosis, and ultimately leads to DDP tolerance [181].

*CircPARD3*, formed by circularization of exon 16–22 of the human PARD3 gene, plays a dual role in cancer development and metastasis [182]. In laryngeal squamous cell carcinoma patients, elevated *circPARD3* levels inhibit autophagy and are associated with malignant progression and poor prognosis. *CircPARD3* acts as a molecular sponge for miR-145-5p, enhancing protein kinase C-iota (PRKCI) expression. PRKCI stimulates the AKT/mTOR pathway, reducing autophagy and causing DDP resistance in laryngeal squamous cell carcinoma [183].

### Oral squamous cell carcinoma (OSCC)

OSCC is a prevalent type of oral cancer. Resistance to DDP limits its efficacy in OSCC treatment. *CircPKD2* (*hsa_circ_0070401*)*,* a crucial circRNA derived from the polycystic kidney disease 2 (PKD2) gene, plays a significant role in influencing autophagy and DDP resistance in OSCC*.* It regulates OSCC proliferation, invasion, and metastasis by modulating the miR‐204‐3p/adenomatous polyposis coli 2 (APC2) axis [185]. Recently, researchers found that increased *circPKD2* levels sequester miR-646, enhancing ATG13 expression. This augmentation of autophagy alleviates DDP resistance in OSCC cells, indicating a promising strategy for overcoming drug resistance in OSCC treatment [186]. Unraveling its role in autophagy regulation provides valuable insights into the mechanisms of DDP resistance, offering potential avenues for therapeutic interventions.

### Prostate cancer (PC)

PC, originating in the prostate gland, is a prevalent cancer in men. Resistance to X-ray therapy poses a significant challenge in its treatment. *CircCCNB2*, a key circRNA, has been identified for its role in promoting autophagy and X-ray resistance in PC. Cyclin B2 (CCNB2) serves as a diagnostic and prognostic marker in various human tumors, including HCC [187], rhabdomyosarcoma [188], TNBC [189], and low-grade glioma [190]. *CircCCNB2*, derived from CCNB2 genomic DNA, is up-regulated in PC patients resistant to radiotherapy. It absorbs miR-30b-5p, elevating KIF18A levels in PC cells. This elevation promotes LC3B and Beclin1 expression, enhancing autophagy activity and leading to X-ray resistance [191]. Understanding its role in autophagy modulation provides essential insights for overcoming X-ray resistance in PC therapy.

## Clinical implications and biomarker potential

The emerging understanding of circRNAs in modulating autophagy has profound clinical implications, particularly in the context of cancer therapeutic resistance. The complex roles of circRNAs in regulating autophagy pathways across diverse cancer types not only shed light on the underlying molecular mechanisms but also present promising avenues for clinical interventions and biomarker development.

### Predictive biomarkers for therapeutic response

Identification of specific circRNAs associated with autophagy dysregulation provides an opportunity to develop predictive biomarkers for therapeutic response. CircRNAs such as *hsa_circ_0072309* in glioblastoma [137] and *circABCB10* in breast cancer [155] exhibit clear correlations with drug sensitivity, suggesting their potential as predictive markers for treatment outcomes. Monitoring the expression levels of these circRNAs could guide clinicians in tailoring therapies based on individual patient profiles, maximizing treatment efficacy.

### Overcoming chemoresistance through targeted circRNA interventions

Targeting dysregulated circRNAs involved in autophagy presents a novel therapeutic approach. Designing small molecules, antisense oligonucleotides, or CRISPR/Cas9-based strategies to modulate circRNA expression could help sensitize cancer cells to conventional treatments. For instance, silencing *circBANP* enhances the effects of X-ray irradiation in colorectal cancer [20], while inhibiting *circINTS4* sensitizes triple-negative breast cancer cells to anthracyclines [158]. These targeted interventions have the potential to reverse chemoresistance and improve patient outcomes.

### Monitoring treatment response and disease progression

Dynamic changes in circRNA expression patterns during treatment could serve as real-time indicators of treatment response and disease progression. Longitudinal monitoring of circRNA profiles, especially those linked to autophagy, might provide valuable insights into treatment efficacy. For example, studies have shown that *circCPM* enhances 5-FU resistance in gastric cancer by promoting autophagy flux [19], and *circ_0006528* activates autophagy and increases paclitaxel resistance in breast cancer by interacting with miR-1299 [156]. Therefore, increased expression of autophagy-promoting circRNAs like *circCPM* and *circ_0006528* could indicate treatment resistance, prompting clinicians to consider alternative therapeutic strategies or combination therapies.

### Non-invasive liquid biopsy biomarkers

CircRNAs have shown stability in body fluids, making them ideal candidates for non-invasive liquid biopsy biomarkers. Circulating circRNAs associated with autophagy dysregulation could be detected in blood, serum, or saliva samples. For example, studies have shown that exosomal *hsa_circ_103801* and *circWDR62* can be detected in serum and serve as non-invasive biomarkers for osteosarcoma/glioma diagnosis and prognosis prediction [76, 98]. Profiling these circRNAs in liquid biopsies might offer a minimally invasive method for diagnosing cancer, predicting therapeutic responses, and monitoring disease recurrence or progression [192].

### Personalized autophagy-targeted therapies

Understanding the circRNA-mediated autophagy networks in individual patients could pave the way for personalized autophagy-targeted therapies. For example, elevated levels of *circABCB10* and *circ_0006528* in breast cancer patients may suggest that these tumors rely on autophagy activation for survival [155, 156]. By analyzing the specific circRNA signatures like *circABCB10* and *circ_0006528* in a patient's tumor, clinicians can tailor treatments to modulate autophagy pathways effectively. This personalized approach holds the potential to enhance treatment responses to paclitaxel and mitigate resistance by blocking circABCB10/ let-7a-5p or circ_0006528/ miR-1299 axes, leading to more effective cancer therapies.

In conclusion, the emerging role of circRNAs in mediating autophagy in cancer therapeutic resistance offers promising avenues for clinical applications. From predictive biomarkers and targeted interventions to non-invasive liquid biopsy markers and personalized therapies, circRNAs have the potential to revolutionize cancer treatment strategies. Continued research and clinical trials focused on circRNA-mediated autophagy regulation are essential to translate these findings into meaningful advancements in cancer patient care.

## Conclusions and perspectives

The burgeoning field of circRNA-mediated autophagy regulation in cancer therapeutic resistance has illuminated novel avenues for understanding the molecular intricacies of drug resistance and has spurred the development of innovative therapeutic strategies. This review summarized the impact and potential mechanisms of circRNAs and autophagy in modulating the resistance of cancer cells to therapy, which provided evident that the complex interplay between circRNAs and autophagy pathways significantly impacts cancer treatment outcomes. The insights gained from studying circRNA-mediated autophagy modulation provide a solid foundation for future research endeavors and therapeutic interventions.

While progress has been made, the complete landscape of circRNAs involved in autophagy regulation remains largely unexplored. Future research should focus on comprehensive profiling of circRNAs across diverse cancer types and stages to identify novel circRNAs and elucidate their roles in autophagy, unveiling new therapeutic targets and biomarkers for predicting treatment responses.

Targeting the unique back-splice sites of aberrant oncogenic circRNAs using antisense oligonucleotides such as siRNA (small interfering RNA) and shRNA (short hairpin RNA), along with CRISPR/Cas13 technology, presents an opportunity to advance cancer treatment [193]. In addition, utilizing tRNA splicing and plasmids containing complementary sequences and specific RBP-binding motifs in the flanking introns to increase the levels of circRNAs with tumor suppressor functions show promise for enhancing their anti-tumor effects [20]. However, ensuring the specificity of circRNA-targeted therapies while minimizing off-target effects is a major challenge, highlighting the need for precise delivery systems that target cancer cells while sparing normal tissue.

Given the conflicting roles of autophagy and circRNA in tumorigenesis, personalized approaches tailored to the circRNA profile of individual patients are essentail. Furthermore, expanding our understanding of circRNA-mediated autophagy regulation beyond the ceRNA mechanism to include other regulatory modes, such as the regulation of RNA modification or translation of new proteins, is crucial for uncovering the precise role of circRNAs in the autophagy pathway [194].

Validating the clinical relevance of autophagy-related circRNAs through large-scale clinical studies and rigorous validation of circRNA-based biomarkers in diverse patient populations is imperative for their widespread adoption in clinical settings. Moreover, integrating different omics data and employing new technologies such as whole-genome screening and single-cell sequencing will provide a more comprehensive understanding of circRNA-mediated autophagy regulation and its impact on cancer treatment resistance.

In conclusion, the emerging field of circRNA-mediated autophagy presents exciting opportunities for cancer therapy. Addressing research gaps and overcoming associated challenges will pave the way for the development of targeted, personalized, and effective therapies, ultimately improving the outcomes and quality of life for cancer patients. Continued interdisciplinary research efforts, collaborative initiatives, and innovative technological advancements are critical to realize the full potential of circRNA-mediated autophagy regulation in cancer therapy.

## Data Availability

The data are available upon request to the author.

## Abbreviations

CircRNA: Circular RNA
MiRNA: MicroRNA
EMT: Epithelial-to-mesenchymal transition
NcRNAs: Non-coding RNAs
RBPs: RNA binding proteins
